# Evolution of Mesenchymal Stem Cell Therapy as an Advanced Therapeutic Medicinal Product (ATMP)—An Indian Perspective

**DOI:** 10.3390/bioengineering9030111

**Published:** 2022-03-07

**Authors:** Sathish Muthu, Madhan Jeyaraman, Moinuddin Basha Kotner, Naveen Jeyaraman, Ramya Lakshmi Rajendran, Shilpa Sharma, Manish Khanna, Sree Naga Sowndary Rajendran, Ji Min Oh, Prakash Gangadaran, Byeong-Cheol Ahn

**Affiliations:** 1 Department of Orthopaedics, Government Medical College and Hospital, Dindigul 624001, India; drsathishmuthu@gmail.com; 2 Indian Stem Cell Study Group, Lucknow 226010, India; moin1503@gmail.com (M.B.K.); naveenjeyaraman@yahoo.com (N.J.); manishvenus@rediffmail.com (M.K.); 3 Department of Biotechnology, School of Engineering and Technology, Sharda University, Greater Noida 201310, India; 4 Department of Orthopaedics, Faculty of Medicine-Sri Lalithambigai Medical College and Hospital, Dr. MGR Educational and Research Institute University, Chennai 600095, India; 5 Fellow in Orthopaedic Rheumatology, Dr. Ram Manohar Lohiya National Law University, Lucknow 226012, India; 6 Fellow in Joint Replacement, Atlas Hospitals, The Tamil Nadu Dr. MGR Medical University, Tiruchirappalli 620002, India; 7 Department of Nuclear Medicine, School of Medicine, Kyungpook National University, Daegu 41944, Korea; ramyag@knu.ac.kr (R.L.R.); ojm0366@knu.ac.kr (J.M.O.); 8 Department of Paediatric Surgery, All India Institute of Medical Sciences, New Delhi 110029, India; drshilpas@gmail.com; 9 Department of Medicine, Sri Venkateshwaraa Medical College Hospital and Research Centre, Puducherry 605107, India; sowndaryasreeraj8@gmail.com; 10 BK21 FOUR KNU Convergence Educational Program of Biomedical Sciences for Creative Future Talents, Department of Biomedical Science, School of Medicine, Kyungpook National University, Daegu 41944, Korea

**Keywords:** ATMP, India, stem cell, mesenchymal stem cell, regulations

## Abstract

Stem cells can be defined as the cells that have the capacity to both self-renew and give rise to differentiated cells. Under the right conditions and signals, depending on their origin and bio-plasticity, stem cells can differentiate into multiple cell lineages and develop into various mature cells. Stem cell therapy is a fast-developing branch of medicine that includes the most innovative regenerative therapies for the restoration of cell and tissue function in individuals with severe diseases. Stem cell research has resulted in the emergence of cell-based therapies for disorders that are resistant to conventional drugs and therapies, and they are considered under the category of an Advanced Therapeutic Medicinal Product (ATMP). The FDA and the European Medicines Agency (EMA) devised a new strategy in 2017 with the aim of unifying the standards for development of ATMPs such that it is easy to exchange information at the international level. In this review, we discuss the evolution of mesenchymal stem cell-based therapy as an ATMP in the global and Indian scenarios, along with the guidelines governing their usage and clinical application of these therapeutics.

## 1. Introduction

Stem cells can be defined as cells that have the ability to self-replicate for an unspecified period [1]. Under the right conditions and signals, depending on their origin and bio-plasticity, stem cells can differentiate into multiple cell lineages and develop into various mature cells [2,3]. Stem cell therapy is a fast-developing branch of medicine that includes the most innovative regenerative therapies for the restoration of cell and tissue function in individuals with severe diseases which do not respond to conventional therapies, including surgeries, radiotherapies and pharmacologic treatments. Stem cell research has resulted in the emergence of cell-based therapies for incurable disorders, and more than 100 illnesses can now be addressed using stem cell therapy [4,5,6,7,8].

In the 1950s, stem cells were employed for the first time as a treatment for marrow aplasia in a mouse model [9]. Of all therapies employed, there are two major categories, namely: (a) hematopoietic stem cell (HSC)-based cell therapy and (b) mesenchymal stem/stromal cells (MSCs)-based cell therapy [10]. HSCs have been extensively researched and used over the last 60 years to treat acute myeloid leukaemia (AML), thalassemia, chronic myeloid leukaemia (CML), sickle cell anaemia, acute lymphoblastic leukaemia (ALL), aplastic anaemia, Hodgkin’s lymphoma, Fanconi anaemia, and other non-Hodgkin’s lymphoma subtypes [7,11].

Since 2012, special interest was developed by researchers on MSCs due to their low immunogenicity compared to HSCs and other cell-based products [4]. MSCs have also been shown to have significant immunological modulation and the ability to modulate the immune system of the host, making them useful in treatment. They offer a wide range of therapeutic uses and are quickly becoming a valuable tool for a variety of pathologies, including cardiac, neurological, and autoimmune illnesses, as well as dermatologic and oncologic disorders [4].

MSCs are currently used in the treatment of graft versus host disease (GVHD), degenerative osteoarthritis, and Burger’s disease [4]. The development of MSC-based products is not only time-consuming, but is also a complex process that includes non-clinical and clinical studies as well as the marketing authorisation imposed by various regulatory agencies, with each product demanding a distinct approach. More than 500 clinical trials on MSC-based products are currently in progress to assess their safety and adverse effects, to define dosages and administration routes, and to determine their efficacy as therapeutic agents at targeted diseases [3,12]. Despite the vast number of clinical trials that have been completed, only ten MSC-based cell therapies have received international approval [13]. This is primarily due to the complexity of these treatments/therapies in terms of tissue/cellular component properties and their legal status as pharmaceuticals.

## 2. Advanced Therapeutic Medicinal Product (ATMP)

According to the European Medicines Agency (EMA), any medical therapies for human use that are based on genes, tissues, or somatic cells should be considered as ATMP [14]. They offer new and innovative possibilities for the management of diseases and injuries. The ATMP family consists of four categories: somatic cell therapy (sCT), gene therapy, tissue engineering (TE), and combined ATMPs (for example, cells embedded in a biodegradable scaffold or matrix) [14,15,16,17].

## 3. MSC as ATMP

MSCs are multipotent stem cells that have a comparable immunophenotypic profile and are found in different anatomical sites such as bone marrow, adipose tissue, the synovium and the umbilical cord [18]. They have the ability to adhere to plastic surfaces, are characterized by having fibroblast morphology, and can differentiate in vitro into osteoblasts, adipocytes and chondrocytes [19,20,21]. MSCs are identified by the expression of cell-specific differentiation markers, namely CD 105, CD 90, CD 73 and low concentrations of MHC-I [21]. MSCs have the ability to migrate to target/injured sites, express their immunomodulator activity, and show paracrine effects [22,23]. With all these features, they are preferred over other types of stem cells as a promising candidate for regenerative medicine and novel therapies.

Despite these advantages, there is a delay in implementation of a theoretical concept of an ATMP into a clinical trial, leading to the approval of a novel treatment. This is probably due to the various challenges posed by the intrinsic nature of ATMPs, which include not only the scientific challenges but also regulatory challenges. MSC-based therapies were classified as an ATMP after the publication of the European Directive 2003/63/EC [24]. Since 2009, the Committee for Advanced Therapies (CAT) under the European Medicines Agency (EMA) has recommended that MSC-based medicines be classified as CT or TE [25]. According to CAT recommendations, if MSCs are substantially manipulated and used to regenerate, replace, or repair human tissue, it should be considered as tissue engineering [24,25]. However, if they are substantially manipulated and are used to treat disease through their immunological, pharmacological, or metabolic actions, then it should be considered as cell therapy [24,25].

## 4. Global Scenario of ATMPs Regulation

In 2009, the first ATMP, approved in the European Union (EU) [24], was Chondrules^®^, a tissue-engineered product for the treatment of cartilage defects. However, in United States (US), one year later, PROVENGE^®^, a somatic cell therapy, was launched for the treatment of prostate cancers [26]. For the clinical use of ATMPs, major regulatory agencies around the world such as the European Medicines Agency (EMA) of the European Union, ref. [17] the Food and Drug Administration (FDA) of United States, ref. [17] the Therapeutic Goods Administration (TGA) of Australia, ref. [27,28] the Medical Devices Agency (PMDA) of Japan, ref. [29] and the Ministry of Food and Drug Safety (MFDS) of the Republic of Korea [30] developed a specific regulatory framework for categorisation and regulation.

Hence, it is vital to know how MSC-based products are categorised and regulated according to their jurisdiction and how to understand which regulatory requirements are involved during their development, including clinical trials and commercialization of the MSC-based product [31,32,33]. The first step in the development of any ATMP is the definition of the product, followed by classification of the product [16,17].

In the European Union, the most relevant subcategory is determined by the active substance’s principal mechanism of action and the declared intended purpose [34]. The cell or tissue product must meet one of the following requirements to be considered “engineered”: (i) the cells or tissues are substantially manipulated or (ii) the cells or tissues are intended to be employed for non-homologous use at donor site [35,36]. If the somatic cells are used for preventing, diagnosing, or treating a disease by immunological, pharmacological or metabolic actions, then they are considered as an sCTMP (somatic cell therapy medicinal product) [17], whereas if the product is used for repair, replacement or regeneration, then it is considered as a TEP (tissue-engineered product) [36].

In the US, the definition and the criteria to include a therapy as somatic cell therapy are: (i) the source should be from autologous, allogeneic or xenogeneic cells; (ii) these cells must have undergone “more than minimal manipulation” (propagated, expanded, selected or pharmacologically treated to alter the biological characteristics); (iii) the therapy is used for preventive, diagnostic or therapeutic purposes [17,37]. Subcategorization of the cellular therapy product does not exist in the US as it does in the EU.

Combined advanced-therapy medicinal products (cATMPs) is a fourth subcategory under the advanced treatments category [17]. In the EU and the US, the product under this subcategory is quite broad and covers medical devices, pharmaceuticals, and biologicals. The EU is governed by two committees: the Committee for Advanced Therapies (CAT) [25] and the Committee for Medicinal Products for Human Use (CHMP) [38]. They are responsible for scientific evaluation and product validation. The classification of ATMPs, the review of their safety, quality, and efficacy, and the monitoring of their scientific developments are the responsibilities of CAT. The core function of CAT is to submit a draft opinion on each ATMP application to the EMA, which helps in the CHMP’s final decision [25,38].

The next step is to obtain market authorization, according to the guidelines of Directive 2001/83/EC and Regulation 726/2004/EC [39]. All ATMPs are assessed via a centralised system for marketing authorization, which ensures the benefit of single evaluation and authorization applicable throughout Europe. There are three ways to obtain marketing authorization: standard, conditional, and exceptional circumstances for extreme cases (e.g., the disease is rare or has an unmeasurable clinical endpoint). Conditional authorization is sought when an innovative therapy used for an unmet medical condition with a low risk-to-benefit ratio is backed up by sufficient clinical data.

The US federal regulatory framework is made up of two important laws: the Public Health Services Act (PHSA) [38,39] and the Federal Food, Drug, and Cosmetic Act (FDCA) [40,41,42]. The Food and Drug Administration (FDA) regulate human medicines such as biological products, pharmaceuticals, and devices [43].

In both the US and the EU, ATMPs are regulated as biological products, because most of them are fulfilled under the description of “drugs”, as mentioned in Section 351 of the PHSA Act [44,45]. An investigational new drug (IND) application is submitted by the applicants to obtain clinical trial approval, along with a biologics licence application (BLA) for market authorization [46,47,48]. Standard, priority review, and accelerated approval are the three types of marketing authorisations. Applications are evaluated within 6 months in priority review compared to 10 months in standard review [46]. Usually, priority review is selected for drugs, which when approved, will result in considerable enhancements in diagnosis, safety or efficacy of treatment, or prevention of a grave disease [49]. If clinical benefit has been established, accelerated approval permits a medicine to be licenced for a serious ailment that satisfies an unmet medical need [50].

To enable faster market authorization of therapies/drugs, breakthrough therapy and fast track designation programs has been developed by the FDA in the US [51], and The PRIority MEdicines (PRIME) designation scheme was developed in the EU [52]. This programme enables for faster marketization approvals aimed at bringing new medicines to the market as quickly as possible. Despite these evolved regulations, both the US and EU are facing challenges regarding the regulation of ATMPs, as they lack experience in this specific medical product group [53,54,55].

## 5. Indian Perspective on Stem Cell Therapies

The definition of stem cell therapy in India is defined as the use of stem cells and stem cell-derived products in all invasive procedures performed by physicians, doctors, and clinicians as part of standard care treatments. Bone marrow transplantation and therapies derived from umbilical cord blood are the most often-used stem cell therapies.

The National Guidelines for Stem Cell Research (NGSCR) was published in 2007 by the Indian Council of Medical Research (ICMR) [56]. They are the main research guidelines in India for conducting clinical research pertinent to stem cells. These guidelines were revised in 2013 and once again in 2017 [57], thereby making them more stringent compared to the initial guidelines published in 2007/2013. 

The NGSCR has clearly elaborated and divided stem cell research into restricted, prohibited, and permitted research [56]. They have also explained what constitutes a minimal, substantial, and massive manipulation. Under the supervision of the Ministry of Health and Family Welfare (MoHFW), the Central Drug Standard Control Organization (CDSCO)/DCGI regulates the isolation, processing, production, and quality of stem cells and stem cell-based products (SCBPs) [58]. In India, SCBPs are regulated as a “New Drug” as per the New Drugs and Clinical Trials (2019), where a new drug includes any stem cell-derived product or gene therapeutic product intended for clinical usage. SCBPs include any stem cell-derived product that is used in the form of a drug intended to be administered to patients.

To initiate a clinical trial related to stem cell therapy, an Institutional Committee for Stem Cell Research (IC-SCR) must be formed (according to the NGSCR, 2017) [56,59]. The purpose of the IC-SCR is to investigate the various aspects of stem cell research such as scientific, technical, ethical, legal, and social issues being an institutional-level self-regulating body. It must therefore be an independently functioning body without any interference, bias, or undue influence. The committee must include at least 11 (medical and nonmedical) representatives in order to function properly [57].

The next step is registration of IC-SCR with the National Apex Committee for Stem Cell Research and Therapy (NAC-SCRT), which consists of an online process and usually takes 8 to 12 months [59]. The NAC-SCRT is an independent apex organisation consisting of professionals from many fields of biomedical research, government agencies, and other stakeholders. It includes a monitoring mechanism at the national level, as it is formed by the Government of India (Department of Health Research) [60]. The institute conducting stem cell research must first apply for a testing license, which allows it to conduct research activities for testing and analysis. The institute has to apply for the No Objection Certificate (NOC) from the CDSCO. This is followed by a joint inspection by CDSCO and the local Food and Drug Agency. The inspection report, in conjunction with the CDSCO’s NOC, can aid in the application for the test license. This licence authorizes the institute to produce stem cells/stem cell derivatives for preclinical testing, including stability studies, efficacy studies, characterization data, and safety studies [57].

The research must be conducted in accordance with Good Manufacturing Practice (GMP)/Good Laboratory Practice (GLP) guidelines [61,62]. This implies that the manufacturing site must adhere to GMP standards, and the testing facility (particularly for safety studies) must be a GLP-accredited laboratory. If larger animals or non-human primates are involved in testing, further approval by the Animal Experiment Control and Supervision Committee is required [63,64]. Clinical Trials (CTs) would be the next stage [65], and the institute must apply for a CT Test License (not the license for sale). The CT protocol is based on the template given in Annexure II of the NGSCR (2017), and the application is submitted to the CDSCO using Form CT-04 or CT-4A, with authorization granted in Form CT-06 [57,66,67].

One more important step before starting the clinical trial is registration of the trial on ctri.gov.in [68]. Before starting the clinical trial, the investigator should study the template (for protocol) and submit a request via Form 44 to the CDSCO office [69]. All trials should comply with the Good Clinical Practise (GCP) guidelines of CDSCO, ref. [70] Schedule Y of the Drug and Cosmetic Act 1940, ref. [67] and the Indian Council of Medical Research (ICMR) Ethical Guidelines [71], which are available on their respective websites.

To monitor any adverse effects during the clinical trial, a data safety monitoring board should be established [72,73]. The main aim of the board is to immediately report all the “side effects” or “adverse events” (SE/AE) by the doctor, investigator, or institute to the IEC and the CDSCO as per the rules of the New Drugs and Clinical Trials (2019).

After confirming all the aspects such as the safety, dosage and efficacy of the stem cell therapy, the next step is market authorization, which, in India, is governed by:

1. CBBTDEC Committee (Cellular Biology Based Therapeutic Drugs Evaluation committee);
2. Technical Committee;
3. Apex Committee.

The CBBTDEC advises the Central Licensing Approval Authority (CLAA) on issues related to clinical trial approval and market authorization for stem cells and stem cell-derived products [65].

The technical committee is in charge of overseeing and monitoring clinical trials in the country. The technical committee’s objective is to oversee clinical trials and provide recommendations to the apex committee for further action [65].

The apex committee is responsible for supervising clinical studies on novel chemical entities in the country. The apex committee’s goal is to review the technical committee’s recommendations on clinical trial approvals and other associated concerns in order to provide suitable direction in the matter [65]. Since the stem cells and SCBPs are associated with unique ethical considerations and obligations, the legal and social concerns that demand additional oversight and expertise is under the control of NAC-SCRT. The IC-SCR’s duty is to primarily approve and monitor the stem cell-based research activities at the institutional level. These “watch-dog” committees ensure the review, approval and monitoring of processes and protocols of stem cell research at accredited laboratories at various levels.

The licensing of indigenous manufacturers of SCBPs requires market authorization from DCGI using Form CT-21 before obtaining a manufacturing license using Form CT-11 from the state licensing authority. In the case of licensing for imported SCBPs, the importer is required to obtain market authorization from DCGI using Form CT-20 before obtaining a registration certificate using Form 41 and import license using Form 10.

Unregulated, dubious stem cell clinics have sprung up all over the world in recent years. Although industrialised countries are increasingly confronted with this issue, it is more prevalent in developing countries, which can be attributed to a lack of policy and regulation. Despite having strict guidelines with respect to stem cell therapy in India, it has become a famous place for unproven stem cell therapies. Despite such clear guidelines, India has about 300 to 500 stem cell clinics. This can be attributed to the fact that violating these guidelines has no legal consequences.

Another significant issue in the stem cell industry is direct-to-consumer advertising, where stem cell clinics attract patients by advertising exaggerated benefits of stem cell therapies. To keep all this in check, there are laws that aim at regulating fraudulent advertising in India. One more important self-regulatory agency is called the Advertising Standards Council of India (ASCI), which plays an import role in monitoring fraudulent advertisements regarding stem cell therapies [74]. The CDSCO suggested a change to the Drugs and Cosmetics Act in April 2018 to classify certain stem cell therapies as drugs [75]. However, the amendment excludes stem cells which are minimally manipulated from the category of drugs, and this omission may defend the use of unproven stem cell treatments. A comparison of the regulatory framework for biological products in the US, Europe, and India is presented in Table 1 and Table 2.

## 6. Future Directives

Although MSC-based ATMPs are considered as drugs, these products require special consideration. As the regulations differ from border to border, the export or import of stem cell therapies becomes difficult. Thus, the FDA and EMA derived a new strategy in 2017 with the aim of unifying the standards for development of ATMPs for an easy exchange of information at the international level.

A number of diseases have shown promising results with stem cell therapy, both in preclinical and clinical trials, including diabetes mellitus, Parkinson’s disease, Crohn’s disease, and a host of haematological disorders. Nevertheless, numerous obstacles such as genetic instability, immune rejection, and ethical concerns need to be solved before they are brought onto the market. Considering India’s rapid development in information technology and biotechnology, development of stem cell therapies that are practical, scalable, and cost-effective is not far from reach.

The future of MSC-based therapy is through cell-free mediators such as exosomes. Several clinical trials are ongoing, with exosomes being used to treat COVID-19 and other neurological diseases [76]. Being a cell-free counterpart to MSCs, exosomes exhibit enhanced safety profiles, thereby making them the therapeutic of choice in many indications.

## 7. Conclusions

The regulation of stem cells in India is challenging due to several factors, which include incoherent rules and regulations and non-statutory guidelines. Another problem with the stem cell governance system in India is the incoordination among the various committees, which itself is a big hurdle to be tackled. The draft amendment to the national stem cell guidelines has highlighted this problem.

ICMR serves as a health advisory organization that develops, coordinates, and promotes research in the biomedical field. In similar fashion, the Department of Biotechnology (DBT) was designated to encourage biotechnology in India but does not enforce any rules onto clinics. As a result, neither the DBT nor the ICMR have the authority to force doctors to follow their recommendations.

Currently, there is no mechanism to monitor the content of social media sites. Hence, regulations for advertising and other rules in India must have a legal basis, and there needs to be assurance that the rules are enforced properly.

As long as the foregoing deficiencies are not addressed, untested stem cell therapies in India will continue to flourish. On a positive note, many research institutes are conducting multiple studies in India in an ethical fashion to extract the best of this treatment for the betterment of the patients. 

## Figures and Tables

**Table 1:** Classification of biological products.

Class	Risk Strategy	Significance	Biological Products
I	Low risk	Products requiring no HCT/P oversight.	Autologous bone marrow-derived stem cells (BMSC). Autologous whole blood and blood-derived products such as PRP. Extracted human products such as collagen.
II	Lower risk	Regulated under Section 361 products with minimal oversight	Allogeneic products. Bone marrow, blood and organ transplants. Amniotic membrane without cells. Bone, cartilage, ligament, cornea, skin, tendon, heart valves, and vascular grafts.
III	High risk	Regulated extensively under Section 351 of HCT/Ps as Biologics and Drugs	Umbilical cord blood. Amniotic tissue cells. Exosomes.

**Table 2:** Regulatory frameworks for biological products in the US, Europe, and India.

Region	US	Europe	India
Regulatory framework	FDA adopts a tiered, risk-based approach contained in a set of regulations called “tissue rules” that regulate HCT/Ps under 21 CFR Part 1271.	In the European Union (EU), stem cells are considered as advanced-therapy medicinal products (ATMPs) and adopts risk-based classification that regulates according to Directive 2009/120 amending Directive 2001/83 for cell-derived medicinal products as per the framework laid down by Regulation (EC) 1394/2007.	Not defined
Regulatory bodies	The Public Health Service Act (PHSA) Food and Drug Cosmetics Act (FDA)	Committee for Advanced Therapies (CAT) within the European Medical Agency (EMEA).	Central Drugs Standard Control Organisation (CDSCO). Indian Council of Medical Research.
Biological Product classification	Low risk Lower risk High risk	Gene Therapy Medicinal Products (GTMP). Somatic Cell Therapy Medicinal Products (CTMP). Tissue Engineered Products (TEP).	As per the current rules of the Drugs and Cosmetics Act, the Ministry of Health and Family Welfare has declared that stem cell and cell-based products are categorised as drugs, which are derived from processed cells including cells or tissues which are processed by means of substantial or more than minimal manipulation with the objective of propagation or differentiation of a cell or tissue, cell activation, or production of a cell line. This includes pharmaceutical, chemical, or enzymatic treatment, altering a biological characteristic, combining with a non-cellular component, or manipulation by genetic engineering, including gene editing and gene modification.

## Data Availability

Not applicable.
